# The Influence of Depression, Anxiety and Cognition on the Treatment Effects of *Ginkgo biloba* Extract EGb 761^®^ in Patients with Tinnitus and Dementia: A Mediation Analysis

**DOI:** 10.3390/jcm10143151

**Published:** 2021-07-16

**Authors:** Petra Brüggemann, Marília Grando Sória, Juliette Brandes-Schramm, Birgit Mazurek

**Affiliations:** 1 Tinnitus Center, Charité–Universitätsmedizin Berlin, 10117 Berlin, Germany; petra.brueggemann@charite.de; 2 Medical Scientific Services, Dr. Willmar Schwabe GmbH & Co. KG, 76227 Karlsruhe, Germany; marilia.soria@schwabe.de (M.G.S.); juliette.brandes-schramm@schwabe.de (J.B.-S.)

**Keywords:** tinnitus, dementia, depression, anxiety, Ginkgo biloba, EGb 761, mediation analysis

## Abstract

Background: Comorbid occurrence of tinnitus and emotional symptoms of anxiety and depression is highly prevalent. The *Ginkgo biloba* extract EGb 761^®^ has been shown to be effective in reducing neuropsychiatric symptoms in patients with dementia and tinnitus. Methods: We performed a mediation analysis to evaluate direct effects of EGb 761^®^ on tinnitus severity, as well as indirect effects mediated by symptoms of depression and anxiety and by changed cognition. We pooled data from subsets of patients suffering from tinnitus that were enrolled in three double-blind, randomized, placebo-controlled clinical trials, which investigated the efficacy of EGb 761^®^ (240 mg/day for 22–24 weeks) in dementia with concomitant neuropsychiatric symptoms. Results: In total, 594 patients suffered from tinnitus (EGb 761^®^, 289; placebo, 305). Direct effects of EGb 761^®^ on tinnitus severity (*p* < 0.001) in patients with mild to moderate dementia were found to represent about 60% of the total effect, whereas the indirect effects (*p* < 0.001) mediated by improvement of anxiety, depression and cognition represented about 40% of the total effect. Conclusions: EGb 761^®^ could be considered as a supporting treatment for tinnitus in elderly patients suffering from dementia, with added benefit in those with symptoms of depression or anxiety.

## 1. Introduction

Tinnitus is a phantom sound sensation that is commonly associated with noise exposure, hearing loss, aging and stress. It is perceived by 10–19% of adults in the general population and approximately by one in three of the elderly [1]. Hearing loss, in turn, has been strongly associated with cognitive impairment, posing a high risk of conversion to dementia 5 to 10 years after its onset [2]. Chronic tinnitus is often accompanied by hearing impairment and the rates of both conditions increase with age [3]. Moreover, tinnitus has been independently associated with cognitive impairment [3,4], therefore it is plausible to expect tinnitus to be a frequent comorbidity in patients diagnosed with dementia. In elderly patients with dementia, Spiegel and colleagues [5] found prevalence rates between 13 and 52% for tinnitus.

On the other hand, symptoms of depression and anxiety are highly prevalent in patients with tinnitus; comorbidity rates between 50 and 90% have been reported from most studies [6]. At the syndrome level (i.e., meeting diagnostic criteria for a mental disorder), Zirke and colleagues [7] found prevalence rates of 37% for affective disorders and of 32% for anxiety disorders. The immediate effect of tinnitus on sleep and the ability to focus and the worry about the underlying causes of disease may render patients more susceptible to psychiatric syndromes. Conversely, patients with or at risk for anxiety and depression may be more inclined to perceive tinnitus and tinnitus-related distress. However, that high comorbidity may also be due to more direct effects. Auditory sensations such as tinnitus and emotions may be processed by the same neuronal networks [8] and, to some extent, explain the high correlations between tinnitus-related distress, depression and anxiety [9,10,11]. Recent findings from neuroimaging studies utilizing connectivity methods suggest that limbic system dysfunction and compromised auditory–limbic interactions may be responsible for the maintenance of tinnitus [12]. The relationship between tinnitus and emotional symptoms or disorders is presumed to be bidirectional. While it appears plausible that the experience of inescapable noise causes depression and anxiety, the relationships between anxiety sensitivity and tinnitus distress [13,14], and mood and tinnitus distress [10], as well as larger improvements in tinnitus severity of patients exhibiting improvement in depression scores [15], suggest that depression and anxiety also have an influence on tinnitus-related distress.

While tinnitus, even in the absence of hearing loss, is associated with cognitive impairment and dementia [2,3,4,16], the role of cognitive impairment in the maintenance and severity of tinnitus is less well understood [2,3,4]. Cholinergic deficits in several brain areas [17], dysfunction of the cognitive control network [18], impaired switching of attention [18], probably due to a dysfunction of the ventral attention network [19], and a general slowing of early stimulus perception [19] may compromise a patient’s ability to habituate to aberrant auditory system activity and to avert the sensation of tinnitus [20].

Current guidelines recommend counseling and cognitive behavioral therapy as first-line treatments, but a few drugs have shown encouraging results in clinical trials and are prescribed in daily practice [21]. Antidepressants may be indicated in patients with concomitant depression, but side effects, including sedation, sexual dysfunction and dry mouth, are common [1,22]. Benzodiazepines may decrease tinnitus-related distress but, beyond side effects like headache, sedation and dizziness, the high risk of dependence prohibits their use in chronic tinnitus [21,23]. Betahistine is fairly well tolerated, with headache and gastrointestinal symptoms being the most common side effects, but its main role is in the treatment of Ménière’s disease [24].

*Ginkgo biloba* special extract EGb 761^®^ (EGb 761^®^ is a registered trademark of Dr. Willmar Schwabe GmbH & Co. KG, Karlsruhe, Germany) is a highly purified dry extract of *Ginkgo biloba* leaves (35–67:1), extraction solvent: acetone 60% (*w*/*w*). The extract is adjusted to 22.0–27.0% Ginkgo flavonoids, calculated as Ginkgo flavone glycosides and 5.4–6.6% terpene lactones consisting of 2.8–3.4% ginkgolides A, B, C and 2.6–3.2% bilobalide, and contains less than 5 ppm ginkgolic acids. A detailed description of these components with their respective molecular structures has been published elsewhere [25].

EGb 761^®^ has been extensively used to treat central nervous system disorders like age-related cognitive impairment and dementia, vestibular and non-vestibular vertigo and tinnitus, as well as peripheral arterial occlusive disease. The mechanism of action behind these therapeutic effects is multifactorial [25], as EGb 761^®^ is not only able to promote neuroprotection and modulate neurotransmission, but also to influence hemorheology to increase blood flow [25]. Animal studies demonstrated that the extract protects against ischemia [26] and hypoxia [27], supports nerve cell energy metabolism, exerts protective effects on mitochondria [28] and shows radical-scavenger activity [29]. These effects may collectively improve brain functions related to tinnitus, especially in the elderly with dementia, who frequently suffer from disorders of vascular etiology and mitochondrial dysfunction [5].

At the neuropsychological level, coping with tinnitus distress seems to be associated with learning and plasticity. In mouse and rat models, EGb 761^®^ was found to increase neuronal plasticity [30] and to influence cholinergic, dopaminergic, noradrenergic and serotonergic transmission [31,32,33,34], which may account for its positive effects on cognition, memory and learning [25], including improvement of auditory discrimination learning [35]. EGb 761^®^ also improves depression- and anxiety-related behaviors and stress reactions in animal models [33,36], which may further contribute to attenuating the distress related to tinnitus.

As mentioned above, tinnitus seems to be frequently associated with cognitive decline and dementia in elderly persons (reviewed by Jafari et al. [3]), therefore the presence and severity of tinnitus and its change under treatment were documented in some of the clinical trials of EGb 761^®^ in patients with dementia (reviewed by Spiegel et al. [5]).

Ten randomized, placebo-controlled clinical trials—summarized by two systematic reviews [5,37]—found moderate improvements in tinnitus loudness and patients’ tinnitus-related distress/annoyance under treatment with EGb 761^®^ in patients with dementia, both when tinnitus was the patients’ main complaint and when tinnitus was associated with dementia. In clinical trials, EGb 761^®^ treatment decreased the severity of anxiety in patients with anxiety disorders [38] or dementia [39] and reduced symptoms of depression in patients with mild cognitive impairment (MCI) or dementia [39,40], irrespective of the absence or presence of tinnitus. Taking into account the findings by Zöger and colleagues [41], that the severity of tinnitus is partially mediated by concomitant depression and anxiety, this raises the question of whether, in patients with tinnitus, EGb 761^®^ directly affects tinnitus severity, or whether it indirectly influences tinnitus severity via anti-depressant and anxiolytic effects.

The objective of this mediation analysis was to assess whether relevant proportions of the effects of EGb 761^®^ on tinnitus severity–assessed by the 11-Point Box Scale in elderly patients with dementia–are mediated by amelioration of symptoms of depression and anxiety, and by improvement of cognition.

## 2. Materials and Methods

### 2.1. Data Sources

For the analyses, we pooled data from three trials that assessed tinnitus and neuropsychiatric symptoms and were published in English in peer-reviewed journals [42,43,44]. The three trials enrolled 1220 patients (EGb 761^®^, 611; placebo, 609) aged 50 years or older with mild to moderate dementia (Alzheimer’s disease, vascular dementia or mixed dementia). Tinnitus severity was assessed by the 11-Point Box Scale [45,46], and depression and anxiety were assessed using the Neuropsychiatric Inventory (NPI) [47]. The 11-Point Box Scale is a type of numeric analogue scale depicting ascending numbers from 0 to 10 in adjacent boxes, with the extreme ends indicating “no tinnitus at all” (0) and “extremely severe tinnitus” (10); in the context of tinnitus, subjectively rated severity most of all represents distress perceived by the patient. The patients were asked to indicate the perceived severity of their tinnitus. The NPI is a clinician interview-based assessment of neuropsychiatric symptoms, including anxiety and depression, in patients with dementia. Following a series of symptom-related interview questions answered by the patients’ caregivers, each symptom is rated by its frequency (1 to 4) and severity (1 to 3), and the symptom-related burden is expressed by a composite score (frequency x severity) ranging from 1 to 12. A score of 0 means that the symptom is absent. The cognitive impairment was assessed by the total score of the SKT Short Cognitive Performance Test [48], a concise cognitive test that consists of 9 subtests to assess attention and memory. Lower scores indicate better performance. Its validity has been demonstrated across Western and Eastern European countries as well as in the United States and Latin American countries, and its total score correlates well with the ability to cope with the demands of everyday life [49]. In all studies, EGb 761^®^ was administered at daily doses of 240 mg, which is the preferred dose for the treatment of dementia [50]; treatment periods of 22 or 24 weeks were chosen following international recommendations for clinical trials in dementia. The 11-Point Box Scale for tinnitus and the NPI were completed together with other dementia-related tests and ratings at screening and baseline visits, at mid-term and end of treatment (in week 22 or 24). In all trials, only a few patients (less than 8%) discontinued the randomized treatment period prematurely [51]. More details are described in the primary publications [42,43,44].

Due to the homogeneity of the trials regarding patient selection, design, treatment period and outcome measures, the data could be pooled, and all analyses are based on pooled data from the three trials. To evaluate direct and indirect effects of EGb 761^®^ on tinnitus severity, the subset of patients suffering from tinnitus (11-Point Box Scale score > 0) (*n* = 594) was selected. In addition, separate analyses were performed for depression and anxiety to investigate the robustness of the results. To assess the proportion of effects of EGb 761^®^ on tinnitus mediated by its relief from depression, the subset of patients who had both tinnitus (11-Point Box Scale score > 0) and depressed mood (item score for depression on the NPI > 0) (*n* = 405) was selected. Likewise, to assess the proportion of effects mediated by the anxiolytic effect of EGb 761^®^, the subset of patients with both tinnitus (11-Point Box Scale score > 0) and anxiety (item score for anxiety on the NPI > 0) (*n* = 466) was analyzed. Furthermore, to investigate the proportion of effects mediated by the treatment effect on cognition, the subset of patients suffering from tinnitus (*n* = 594) was analyzed.

### 2.2. Statistical Analyses

The post hoc mediation analyses were based on subsets of the full-analysis data sets of the clinical trials.

Statistical mediation models were used to investigate direct and indirect effects of EGb 761^®^ on tinnitus severity in patients with dementia. Mediation models can be applied to investigate the relationship between an independent variable and a dependent variable through the introduction of one or more mediator variable(s) [52]. The effects of EGb 761^®^ (independent variable) on tinnitus severity (dependent variable) mediated by the effects of reduced depression, improved anxiety symptoms or improved cognition (mediator variables) were described using a mediation model with three mediators. Furthermore, separate analyses including one mediator were performed.

Treatment effects on tinnitus severity were evaluated using the changes in the tinnitus score between baseline and the end of treatment. The change in the NPI depression item score between baseline and end of treatment was used to describe the anti-depressive effects, the change in the NPI anxiety item score was used to describe the anxiolytic effects and the change in the SKT total score was used to assess the effect on cognition. Missing values were replaced by LOCF as performed for the original analyses of these trials.

The following linear regression models were used to estimate and test the total, direct and indirect effects in a mediation model with three mediators.

Model 1 Y = i1 + cX + ε1(1)

Model 2 Y = i2 + c’X + β1M1 + β2M2 + β3M3 + ε2(2)

**Figure 1:**
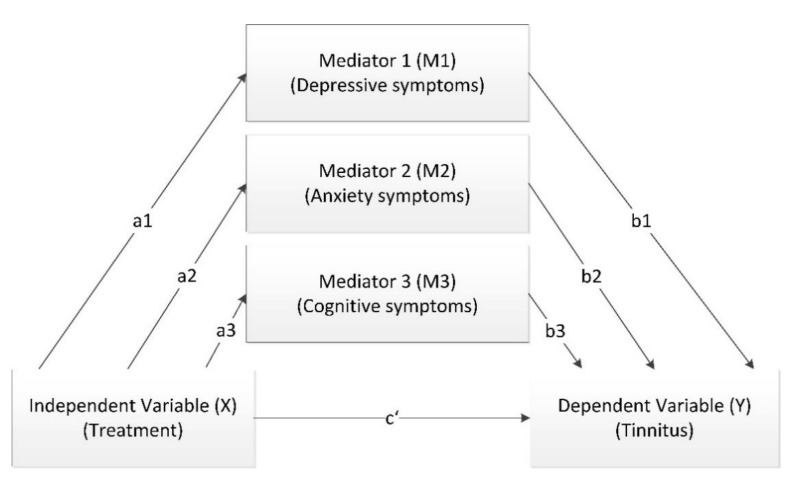
Mediation model with three mediators (please refer to text for details).

Model 3 Mj = i3j + αjX + ε3j(3)

where Y is the outcome variable (change in the tinnitus score between baseline and end of treatment), X is the independent variable (treatment), Mj are the mediators (j = 1, 2, 3 change in NPI item scores and SKT total score between baseline and end of treatment), c is the total effect of the independent variable on the outcome, c’ is the coefficient relating the independent variable to the outcome (direct effect) adjusted for the effects of the mediators, ε1, ε2 and ε3j are the unexplained variances, i1, i2 and i3j are the intercepts (j = 1, 2, 3). The indirect effects can be calculated by multiplying the regression coefficients αj and βj. The combined indirect effect can be estimated by adding the indirect effects. The mediation model with three mediators is illustrated in Figure 1.

Sobel’s test [53] was used to investigate the extent to which the mediators (anti-depressive/anxiolytic/cognitive effect) contribute to the total effect of the independent variable (treatment) on the outcome variable (changes of tinnitus severity). The test of Sobel assumes normality and no measurement error. In order to investigate the robustness of the results, the method of Hayes [54], which estimates the indirect effects and corresponding 95% confidence intervals using bootstrapping, was also applied. This method does not assume normally distributed data.

The direct and indirect effects of the independent variable (treatment) on the outcome variable (changes of tinnitus severity) were estimated as percentages of the total effect.

A *p*-value less than 0.05 was considered statistically significant in a descriptive sense. All analyses were performed using SAS Version 9.4 (SAS Institute INC, Cary NC, USA) and Windows 10 Enterprise (Microsoft Corporation, Redmond, WA, USA).

## 3. Results

Altogether, 1220 patients were enrolled in the three trials and randomly assigned to receive EGb 761^®^ (*n* = 611) or placebo (*n* = 609). Of these, 594 patients (EGb 761^®^, 289; placebo, 305) suffered from tinnitus when the treatment started. For 518 of these patients, concomitant depression or anxiety symptoms were documented, 353 subjects suffered from both depression and anxiety, 405 patients suffered from depression and tinnitus, 466 from anxiety and tinnitus. The subsequent analyses are based on 594 patients suffering from tinnitus (with or without depressive symptoms or anxiety).

Demographic data, baseline scores and changes during treatment are shown in Table 1. Baseline data (like age and sex) were comparable between the treatment groups within the trials. In all trials, treatment group mean baseline scores of tinnitus severity, depression, anxiety and cognition were similar and the mean reductions in tinnitus severity, depression, anxiety and cognitive impairment from baseline were more pronounced for patients of the EGb 761^®^ group compared to the placebo group. Statistically significant treatment effects could be observed in the subgroup of patients suffering from tinnitus in trials A and B with respect to tinnitus severity, depression, anxiety and cognitive impairment. In trial C, a statistically significant treatment effect on cognition was observed, and small, not statistically significant differences favoring EGb 761^®^ were observed for the other symptoms. Compared to the other trials, reductions in tinnitus, depression and anxiety scores of patients treated with EGb 761^®^ are similar while changes in the placebo group are slightly more favorable in trial C. The single trials were not planned and powered to show statistically significant treatment effects in the subgroups of patients suffering from tinnitus. The data of these clinical trials could be pooled since the demographic characteristics and the baseline scores of the outcomes are well balanced across treatment groups.

At baseline, anxious symptoms showed a statistically significant effect on tinnitus severity, while the effects of depressive symptoms and cognitive impairment on tinnitus were not statistically significant.

Although mean scores of tinnitus were low to moderate (about 3 points on the 11-Point Box Scale) at baseline, a significant superiority of EGb 761^®^ compared to placebo could be observed with respect to the reduction in the scores until the end of the treatment.

The results of the mediation analysis including three mediators are presented in Table 2. The mediation analysis shows that the statistically significant direct effect of EGb 761^®^ on tinnitus (*p* < 0.001) represents 58.5% of the total effect, and that the likewise statistically significant indirect effects mediated by reduced depressive symptoms, improved anxiety symptoms and improved cognition (*p* ≤ 0.004 for all indirect effects) overall represent 41.5% of the total effect. The analysis using the bootstrap method supported these results (Table 3).

In addition, three models with one mediator each were applied (data not shown). The analyses based on these models indicate that the changes in depression severity/anxiety severity/cognitive impairment under EGb 761^®^ treatment explains part of the improvement of tinnitus, whereas the largest proportion of the observed improvement in tinnitus was due to a direct effect of EGb 761^®^. The results obtained from these models supported the results based on the model with three mediators.

## 4. Discussion

Although mean scores of tinnitus at baseline were low, a statistically significant and clinically meaningful superiority of EGb 761^®^ compared to placebo could be observed with respect to reduction in the scores at the end of the treatment in patients with dementia. Changes in tinnitus severity, cognition, anxiety and depressive symptoms were correlated.

The mediation analysis showed that EGb 761^®^ treatment decreased tinnitus severity in a cohort of dementia patients and that there is an additional effect on tinnitus severity mediated by the improvement in depression, anxiety and cognition by EGb 761^®^. The results obtained from a model with three mediators were supported by the analyses based on three models with one mediator each.

The mediating effect of improved psychological symptoms is in line with findings reported by Procházková et al. [55] from a study in non-demented patients. They observed a tendency towards larger improvements in tinnitus distress, loudness and annoyance in a subgroup of depressed patients treated with EGb 761^®^.

The high prevalence of tinnitus in patients with dementia is not surprising. Hearing loss is often associated with tinnitus, and patients with hearing loss have an increased risk of developing dementia and tinnitus [3,56,57]. Moreover, tinnitus itself, even in the absence of hearing loss, affects a variety of cognitive functions [4,16] and may thus contribute to the development of dementia. This ties in with findings by other research groups who reported that functional disturbances in various neuronal networks (e.g., in the thalamus, limbic areas and frontal circuits) and structural changes, as well as decreased neurogenesis in the hippocampus, are associated with tinnitus as well as with Alzheimer’s disease and other dementias [8,58].

A strength of this mediation analysis is the pooling of data from multiple studies, leading to the inclusion of a large number of participants. Data pooling was possible since all three included trials used very similar inclusion criteria and outcome measures. The caregiver interview-based NPI items for anxiety and depression do not permit a diagnosis at the syndrome level or a distinction between syndromal or sub-syndromal severity, but the scores correlate well with scores on widely used clinician rating scales for anxiety [47,59] or depression [47,60]. Concerns may be raised regarding the reliability of tinnitus ratings by patients with mild to moderate dementia. We nevertheless consider the self-assessments as reliable. The assessments of tinnitus severity and the changes during the treatment period may be less precise than in cognitively healthy persons. It is, however, highly unlikely that a consistent pattern of superiority of EGb 761^®^ over placebo would result from three large, independent, double-blind trials if the assessments had been unreliable and largely driven by chance.

Another limitation of the analyses may be that a mediation effect in the opposite direction, i.e., a reduction in depression and anxiety scores or an improvement in cognition as a consequence of decreased tinnitus severity, cannot be ruled out with certainty. This was not the question underlying the present study but might challenge the validity of our model. On the other hand, an influence of depression and anxiety on tinnitus severity, the basic assumption underlying our analyses, is supported by a number of independent studies (e.g., [10,13,14,15]). It also seems plausible that, by enhancing hippocampal neurogenesis, as shown in animal models [61], and by improving cognitive functioning, as shown in patients with dementia [51], EGb 761^®^ may improve tinnitus distress [58]. Our finding of a strong mediating effect of cognition on tinnitus distress could strengthen the hypothesis that cognitive impairment may contribute to the perception and severity of tinnitus [20]. To fully disentangle the relationship between tinnitus, depression, anxiety and cognition, more research is warranted.

Although the assumption of causality for the effect of the mediator on the dependent variable appears reasonable, causality typically cannot be tested in this type of analysis [62]. A more realistic approach is to incorporate additional information from prior research.

Since many trials of pharmacological and non-pharmacological treatments of tinnitus have failed at the group level, presumably because of considerable heterogeneity of the condition, attempts have been made to identify groups of patients likely to respond to a variety of individualized treatments [63,64]. In accordance with our findings, EGb 761^®^ may be considered for the individualized treatment of elderly patients with dementia and tinnitus showing symptoms of depression and/or anxiety.

Taking into account the role of EGb 761^®^ as an anti-dementia agent [51,65] and the recently reported relationship between tinnitus and cognition [3,19,66], further exploration of the potential influence of tinnitus on the severity of cognitive impairment in dementia appears worthwhile. This may provide additional clues for the identification of molecular mechanisms involved in conveying the effects of EGb 761^®^ on tinnitus in patients with dementia.

## 5. Conclusions

In our mediation analysis, based on the pooled data from three randomized, placebo-controlled trials, we found (1) indirect effects of EGb 761^®^ on tinnitus severity, which are mediated by attenuation of symptoms of depression and anxiety and by improved cognition, and (2) a direct effect on tinnitus severity in patients with dementia.

EGb 761^®^ may therefore be considered a treatment for tinnitus in elderly patients with dementia, with added benefit in patients with symptoms of depression or anxiety.

## Figures and Tables

**Table 1:** Demographic data, baseline scores, changes during treatment and correlations of patients who had tinnitus at baseline (data from three trials, absolute and relative frequency and *p*-value of the two-sided χ^2^ test or mean ± standard deviation, *p*-value of the two-sided *t*-test and Pearson correlation coefficient, respectively).

					Baseline Scores				Change Scores			
Trial ^1^	Treatment	N	Female (%)	Age (Years)	Tinnitus	Depression	Anxiety	Cognition	Tinnitus	Depression	Anxiety	Cognition
A	EGb 761^®^	102	76 (74.5%)	64.8 (8.5)	4.0 (1.6)	2.0 (1.6)	3.4 (2.6)	16.5 (3.5)	−2.1 (1.7)	−0.9 (1.4)	−1.3 (1.8)	−3.3 (2.2)
	Placebo	104	76 (73.1%)	63.7 (8.6)	3.9 (1.4)	2.1 (1.7)	3.8 (2.4)	16.0 (3.5)	−0.2 (1.0)	0.4 (1.5)	0.0 (1.9)	1.3 (2.1)
	*p*-value		0.815	0.378	0.527	0.550	0.2764	0.265	<0.001	<0.001	<0.001	<0.001
B	EGb 761^®^	90	65 (72.2%)	65.0 (9.7)	2.9 (1.5)	1.5 (1.8)	2.2 (2.1)	16.6 (3.8)	−1.1 (1.2)	−0.7 (1.4)	−0.6 (1.6)	−1.9 (2.8)
	Placebo	107	73 (68.2%)	65.2 (9.2)	2.9 (1.5)	1.7 (1.9)	2.4 (2.2)	17.1 (3.5)	−0.1 (0.9)	−0.1 (1.3)	−0.0 (1.8)	0.4 (2.7)
	*p*-value		0.542	0.8907	0.977	0.473	0.501	0.336	<0.001	0.002	0.014	<0.001
C	EGb 761^®^	97	68 (70.1%)	65.9 (8.6)	2.9 (1.6)	2.9 (2.4)	3.5 (2.3)	15.2 (3.9)	−0.8 (1.4)	−0.6 (2.0)	−1.1 (2.0)	−2.1 (3.0)
	Placebo	94	65 (69.1%)	66.7 (9.7)	3.0 (1.4)	2.9 (2.1)	3.4 (2.4)	16.1 (3.9)	−0.7 (1.2)	−0.4 (1.8)	−0.7 (1.6)	−0.3 (2.8)
	*p*-value		0.886	0.578	0.576	0.931	0.698	0.138	0.440	0.464	0.1853	<0.001
Overall	EGb 761^®^	289	209 (72.3%)	65.2 (8.9)	3.3 (1.7)	2.1 (2.0)	3.1 (2.4)	16.1 (3.8)	−1.4 (1.6)	−0.7 (1.6)	−1.0 (1.8)	−2.5 (2.7)
	Placebo	305	214 (70.2%)	65.1 (9.2)	3.3 (1.5)	2.2 (2.0)	3.2 (2.4)	16.4 (3.7)	−0.3 (1.1)	−0.0 (1.5)	−0.2 (1.8)	0.5 (2.6)
	*p*-value		0.562	0.906	0.864	0.634	0.582	0.352	<0.001	<0.001	<0.001	<0.001
Influence of baseline depression/anxiety/cognition on tinnitus severity ^2^						*p* = 0.172	*p* = 0.006	*p* = 0.715				
Correlation between tinnitus score change and depression/anxiety/cognition score change during treatment									EGb 761^®^	0.234	0.202	0.187
									Placebo	0.241	0.204	0.339

^1^ Subgroups of patients suffering from tinnitus in trials of Napryeyenko et al. [42] (A), Ihl et al. [43] (B) and Herrschaft et al. [44] (C). ^2^ Multiple regression to investigate the influence of baseline depression, anxiety and cognition on tinnitus severity.

**Table 2:** Results of the analysis with three mediators based on pooled data from three trials (subset of patients with tinnitus at baseline, *n* = 594, full analysis set); parameter estimate, standard error (SE) and *p*-value.

Effect	Estimate	SE	*p*-Value
a1: treatment effect on depression (model 3)	0.7112	0.130	<0.001 ^(1)^
a2: treatment effect on anxiety (model 3)	0.8051	0.148	<0.001 ^(1)^
a3: treatment effect on cognition (model 3)	2.9287	0.219	<0.001 ^(1)^
b1: mediator effect (depression) on tinnitus (model 2)	0.1234	0.035	0.001 ^(1)^
b2: mediator effect (anxiety) on tinnitus (model 2)	0.1031	0.030	0.001 ^(1)^
b3: mediator effect (cognition) on tinnitus (model 2)	0.0912	0.0204	<0.001 ^(^^1)^
c’: direct effect (model 2)	0.6169	0.120	<0.001 ^(1)^
c: total effect (model 1)	1.0549	0.110	<0.001 ^(1)^
a1 × b1: indirect effect mediated by reduced depression	0.0877	0.030	0.004 ^(2)^
a2 × b2: indirect effect mediated by reduced anxiety	0.0830	0.029	0.004 ^(^^2)^
a3 × b3: indirect effect mediated by improved cognition	0.2672	0.063	<0.001 ^(1)^

^(1)^ Two-sided *t*-test *p*-value; ^(2)^ *p*-value of Sobel’s test.

**Table 3:** Estimation of indirect effects using the bootstrap method—model with three mediators based on pooled data from three trials (subset of patients with tinnitus at baseline, full analysis set); parameter estimate, standard error (SE) and 95% confidence interval (CI).

Effect	Estimate	SE	95% CI
a1 × b1 + a2 × b2 + a3 × b3: indirect effects (combined)	0.4380	0.077	(0.2899; 0.5952)
a1 × b1: indirect effect mediated by reduced depression	0.0877	0.032	(0.0319; 0.1590)
a2 × b2: indirect effect mediated by reduced anxiety	0.0830	0.032	(0.0282; 0.1523)
a3 × b3: indirect effect mediated by improved cognition	0.2672	0.074	(0.1241; 0.4186)

## Data Availability

Due to ethical reasons and in terms of data protection law, raw data cannot be shared as requested. To the extent permitted by law, trial data required for validation purposes are already disclosed in result reports on corresponding databases. All relevant data are within the paper.
